# Purposeful use of multimodality imaging in the diagnosis of caseous mitral annular calcification: a case series report

**DOI:** 10.1186/s12880-021-00725-x

**Published:** 2022-01-06

**Authors:** Krunoslav Michael Sveric, Ivan Platzek, Elena Golgor, Ralf-Thorsten Hoffmann, Axel Linke, Stefanie Jellinghaus

**Affiliations:** 1 Department of Internal Medicine and Cardiology, Herzzentrum Dresden, Technische Universität Dresden, Fetscherstr. 76, 01307 Dresden, Germany; 2 Insitute and Polyclinic for Diagnostic and Interventional Radiology, Dresden University Hospital, Technische Universität Dresden, Fetscherstr. 74, 01307 Dresden, Germany

**Keywords:** Caseous, Mitral annular calcification, Multimodality imaging

## Abstract

**Background:**

Caseous mitral annular calcification (CMAC) is a rare liquefactive variant of mitral annular calcification (MAC) and superficially mimics a cardiac vegetation or abscess. CMAC is viewed as a benign condition of MAC, while MAC has clinical implications for patients’ lives. Correctly diagnosing CMAC is essential in order to avoid unnecessary interventions, cardiac surgery or even psychological suffering for the patient.

**Case presentation:**

We report on 6 patients with suspected intra-cardiac masses of the mitral annulus that were referred to our institution for further clarification. A definitive diagnosis of CMAC was achieved by combining echocardiography (Echo), cardiac magnetic resonance imaging (MRI) and cardiac computed tomography (CT) for these patients. Echo assessed the mass itself and possible interactions with the mitral valve. MRI was useful in differentiating the tissue from other benign or malign neoplasms. CT revealed the typical structure of CMAC with a “soft” liquefied centre and an outer capsule with calcification.

**Conclusion:**

CMAC is a rare condition, and most clinicians and even radiologists are not familiar with it. CMAC can be mistaken for an intra-cardiac tumour, thombus, vegetation, or abscess. Non-invasive multimodality imaging (i.e. Echo, MRI, and CT) helps to establish a definitive diagnosis of CMAC and avoid unnecessary interventions especially in uncertain cases.

## Background

Calcification of the mitral annulus (MAC) is a common finding particularly in elderly patients. MAC is associated with renal and cardiovascular morbidity [1]. In contrast, caseous mitral annular calcification (CMAC) is generally regarded as a benign entity of MAC. However, recent studies reported that CMAC is associated with an increased risk of stroke and potential conduction abnormalities due to spontaneous fistulisation [2–4]. CMAC is rare (< 1%), often manifests as a local periannular mass, and rarely interacts with the function of the mitral valve [5, 6]. A reliable CMAC diagnosis is thus important in order to avoid unnecessary interventions, cardiac surgery or even psychological suffering for the patient. Here we present 6 unclear cases of CMAC that were eventually diagnosed through the use of non-invasive multimodal imaging techniques such as standard transthoracic echocardiography (Echo), cardiac magnetic resonance imaging (MRI) and biphasic iodine contrast-enhanced electrocardiographically gated computed tomography (CT).

## Case presentation

### Clinical characteristics

**Fig. 1:**
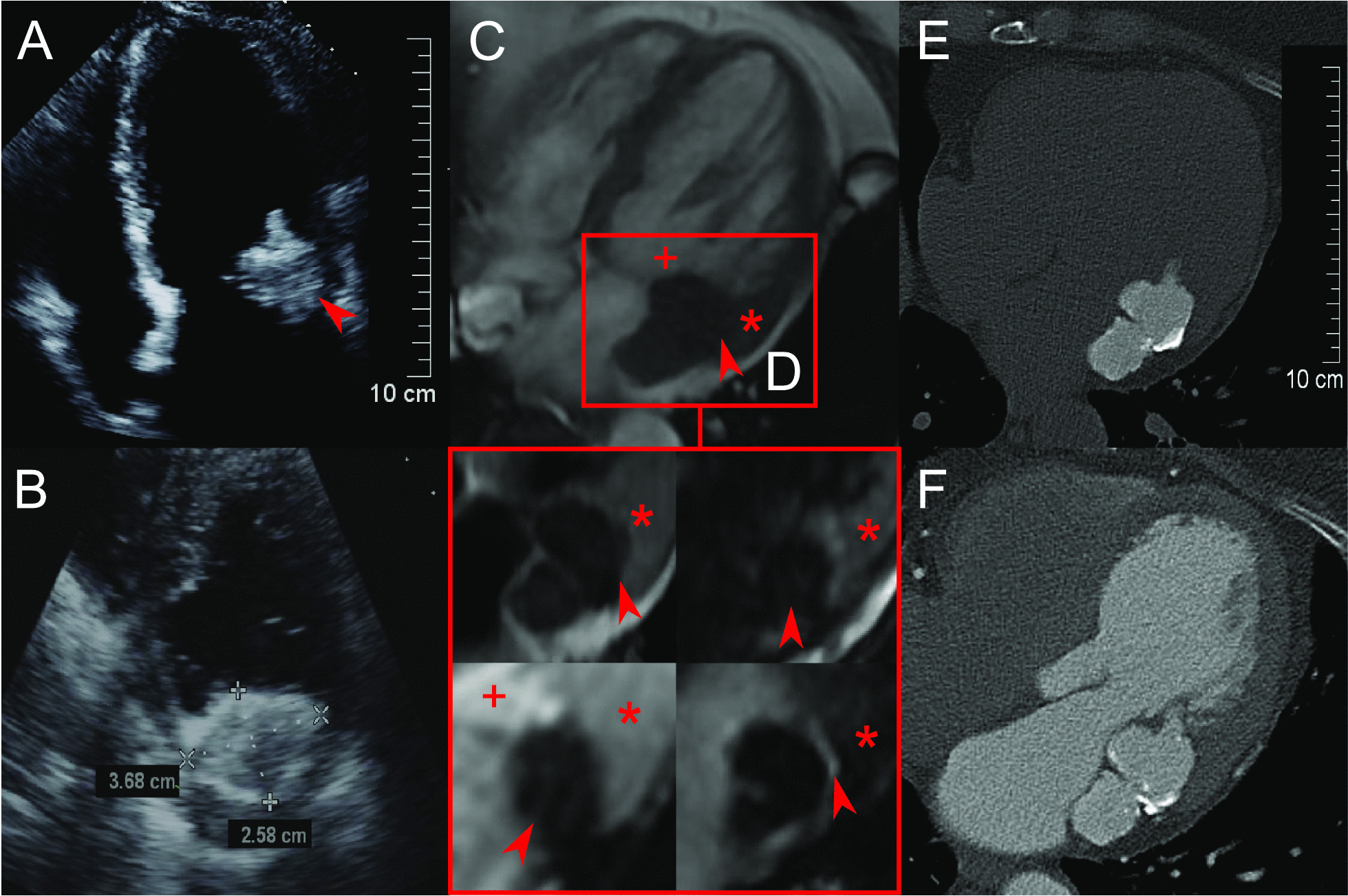
Exemplary characteristics of CMAC (case 6). Echo: apical long axis view **A** with the hyper-echoic mass (arrow) at the lateral mitral valve annulus with acoustic shadowing; short-axis view **B** with a blurred demarcation of the hyper-echoic capsule and hypo-echoic core. MRI: cardiac 4-chamber view in steady state free precision sequence **C** with the iso-intense mass (arrow) to the adjacent myocardium (star), but with a clear hypo-intense demarcation from intracavitary blood (plus sign); T1-weighted sequence with the hypo-intense mass (arrow) and less intense core in T2-weighted sequence (arrow) compared to the myocardium (star) (**D**; top left and top right); first pass perfusion with low signals of vascularisation compared to myocardium (star) and intraventricular blood (plus sign) (**D**; bottom left); late gadolinium enhancement with very low signal in the core, but peripheral bright border (arrow) (**D**; bottom right); CT: precontrast hyperattenuation of the CMAC mass with peripheral calcifications (**E**) and no accumulation of contrast agent (**D**). *CMAC* caseous mitral annular calcification, *Echo* transthoracic echocardiography, *MRI* cardiac magnetic resonance imaging, *CT* cardiac computed tomography

Six out-patients were initially referred to our institution for further evaluation of echocardiographically detected intra-cardiac masses at the mitral annulus. The main clinical characteristics of the patients are summarised in Table 1. Interestingly, the female sex was predominant (5 of 6) with a median age of 79 years, and with cardiovascular risk factors such as arterial hypertension, dyslipidaemia and reduced renal function. Three patients had previously suffered ischemic strokes or transitory ischemic attacks of unknown etiology. It was thus necessary to eliminate the possibility of thrombus. In addition, echocardiographic windows were impaired and did not allow a definitive diagnosis of CMAC. In cases 1 and 4, severe calcification of the aortic valve was already suspected by the referring hospital. Careful diagnosis was therefore imperative before treatment of severe aortic stenosis in these frail patients. Furthermore, two patients (case 2 and 5) had slightly elevated inflammatory markers (c-reactive protein 20 and 25 mg/l), but without leukocytosis (4–10 × 10^9^/l). The one male patient (case 6), however, had lived in a tuberculosis incidence area for several years for professional reasons. A tuberculin test was not conclusive due to vaccination in childhood. These uncertain cases therefore persuaded us to perform a successive three-step approach with Echo, MRI and CT for further clarification. The typical imaging characteristics of CMAC are illustrated as an example in Fig. 1.

**Table 1:** Main clinical characteristics

Case	Sex	Age (years)	BMI	GFR (ml/min)	LDL level (mmol/l)	Statin therapy	aHT	History of stroke/TIA	MR	LVEF (%)
1	F	89	24	31	3.1	y	y	y	II	63
2	F	74	29	54	2.6	y	y	n	II	64
3	F	75	23	40	3.4	y	y	y	I–II	40
4	F	91	23	53	4.2	y	y	y	I-II	65
5	F	79	35	51	2.7	y	y	n	I	61
6	M	62	31	72	3.8	n	y	n	I	65

F, female; M, male; BMI, body mass index; GFR, glomerular filtration rate; LDL, low density lipoprotein; ahT, arterial hypertension; TIA, transitory ischemic attack; MR, mitral valve regurgitation; LVEF, left ventricular ejection fraction; y, yes; n, no

### Echocardiography findings

First, CMAC typically appeared in Echo as a mostly spherical, static and echo-dense (i.e. hyper-echoic) mass with smooth borders in the posterior-lateral periannular region. It contained one or several central hypo-echoic areas (Fig. 1A, B). CMAC and the adjacent annular calcification showed some degree of acoustic shadowing, which prevented precise discrimination of the masses in the left atrium or the myocardium.

### Magnetic resonance imaging findings

Second, the masses appeared larger in MRI than in Echo, but clear differentiation of the CMAC mass from the adjacent myocardium was impaired in the standard steady-state free precision “cine” sequences (Fig. 1C). In contrast, T1- and T2 weighted MRI sequences provided a better depiction of the architecture and morphology of the mass (Fig. 1D, top left, top right). Furthermore, the extent of the mass was more easily distinguished from the adjacent myocardium through its hypo-intensity (i.e. low signal intensity) in these sequences. There were no contrast first pass perfusion effects, and no significant late gadolinium enhancement at the core of the CMAC mass (Fig. 1D, bottom left, bottom right). Absence of vascularisation or central necrosis distinguishes it from benign or malign tumours. A description of how multimodality imaging can differentiate CMAC from other intracardiac masses will follow in the discussion section. Additionally, a thin peripheral ring of late gadolinium enhancement indicated the typical CMAC capsule in MRI, but MRI had the disadvantage of not disclosing calcified areas as precisely as CT.

### Computed tomography findings

CT scans revealed the typical demarcation of the hyper-dense structure of the “amorphic” and avascular centre without contrast enhancement from the fibrotic capsule with small irregular calcifications (Fig. 1E, F).

### Further implications of multimodality imaging

**Fig. 2:**
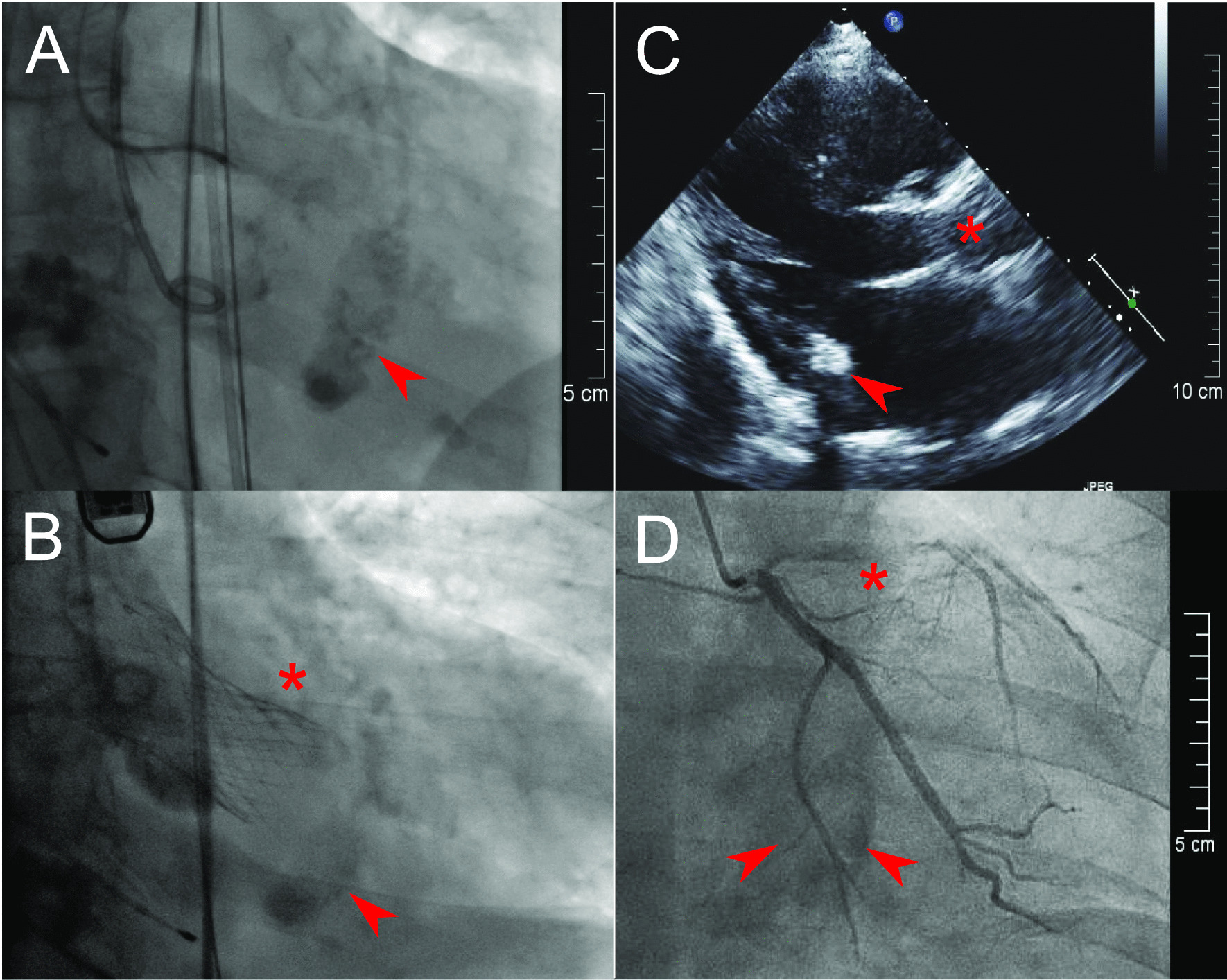
Before (**A**) and after (**B**) transcatheter aortic valve prothesis (star) for severe aortic stenosis with CMAC (arrow) at the inferior mitral valve annulus in case 4. Echocardiographic 1 year follow-up visit after transcatheter aortic valve replacement of case 1 (**C**) with the aortic prosthesis (star) and CMAC mass at posterior mitral valve annulus (star). Percutaneous coronary intervention of the left anterior descending artery (star) and the “shadow” of CMAC (arrow) at the bottom image of case 3 (**D**). *CMAC* caseous mitral annular calcification

Nevertheless, the systematic use of these three imaging modalities gave us not only a definitive diagnosis of CMAC, but also information about important “bystander” diagnoses. For example, due to high frailty in cases 1 and 4, CT played a key role in planning the transcatheter aortic valve replacement for severe aortic stenosis initially diagnosed by Echo (Fig. 2A–C). Furthermore, Echo identified an impaired left ventricular function with regional anterior hypokinesia in case 3. MRI verified this as a reversible perfusion defect through stress perfusion sequences with application of adenosis and led to a successful percutaneous coronary intervention of a relevant stenosis in the left anterior descending artery (Fig. 2D). It is important to note that MRI stress sequences were performed only in this one particular case (#3) with suspected myocardial ischemia. In general, the first pass effect in MRI is sufficient to assess the vascularisation of the tumour. In cases 1, 3 and 4, a history of stroke/transitory ischemic attacks was associated with atrial fibrillation without adequate anticoagulation, which we detected by means of long-term electrocardiograms. It is worth mentioning that after comprehensive evaluation of all cases by the heart team, no patient was referred to cardiac surgery for CMAC.

## Discussion

CMAC is a rare variant of the more common MAC [6]. The most challenging aspect of CMAC is its misdiagnosis as a myocardial abscess, cardiac neoplasm, or even thrombus. Echo serves as the first line imaging tool for assessing the mass itself and possible interactions with the mitral valve. In straightforward cases, when CMAC is detected incidentally, Echo identifies the typical characteristics of CMAC: its smooth border; its typical location in the postero-lateral annular region of the mitral valve; its overall echo-dense structure with hypo-echoic central areas. Hence, no further work up is needed in general [5, 7]. Indeed, a myocardial abscess in early stages can be detected by Echo due to its rather non-homogeneous appearance, an echo-free space or due to blood flow. However, in later stages, when these abscesses are consolidated and calcified a clear differentiation from CMAC is very difficult. In uncertain cases, such as where the acoustic window is impaired, when non-specific elevation of inflammation markers are present, when anamnesis suggests an underlying malignancy, or, as in our case 6, where a history of tuberculosis cannot be ruled out, systematic use of MRI or CT is helpful. Furthermore, differentiation from smaller, less mobile myxoma can be cumbersome, since myxoma mostly appear in the left atrium. Cardiac MRI offers the unique opportunity to differentiate cardiac masses based on specific signal intensities, contrast first pass perfusion, and late gadolinium enhancement (Fig. 1D). T1- and T2-weighted sequences are useful in assessing the size and extent of the cardiac mass, due to its different signal intensity relative to the myocardium [8]. Hypo-, iso- or hyper-intensity indicates the specific fat and water content of the tissue in question. This method allows differentiation from various tumours, such as myxoma and lipoma [8, 9]. CMAC, for instance, shows a hypo-intense core in T1- and T2-weighted sequences, while myxoma and lipoma exhibit central hyper-intensities in T2-weighted sequences, due to their mucinous or fatty stroma. Similarly, myocardial abscesses display a central hyper-intensity with a hypo-intense layer, although these characteristics vary by abscess stage and type. Although myocardial tuberculosis is extremely rare and is supposed to manifest in pericardial or prevascular localisations, we could not rule out a tuberculoma in case 3. In general, T1- and T2-weighted MRI sequences reveal non-homogeneous hyper-intensities of the tuberculous granuloma, but these imaging features are not specific [10]. A contrast first pass perfusion sequence uses the same assets as myocardial ischemia detection, and reflects the amount of vascularisation of the cardiac mass. This specific MRI method is helpful in differentiating malign from benign masses, for instance, between Sarcoma and thrombi [11, 12]. CMAC is a mass without vascularisation, hence no early accumulation of gadolinium is expected. Furthermore, late gadolinium enhancement can be detected more often in malign tumours indicating tissue necrosis, but is seldom found in benign neoplasms and very rarely in thrombi [13]. Typically, CMAC does not show signs of late gadolinium accumulation, due to the absence of necrotic tissue. Thrombi commonly share the same feature in these MRI sequences, differentiation on the basis of the characteristics may remain challenging. Myxomas, for instance, show partial signs of vascularisation or necrosis in MRI, which makes it difficult to further differentiate them from malign masses [12]. If an MRI is inconclusive further imaging steps may be necessary. Although MRI is helpful in tissue characterisation, it is prone to artefacts during long measurement times caused by inadequate breath holds, cardiac motion and arrhythmia. As well as having limited availability, cardiac MRI in our view requires more laborious planning than CT. Cardiac CT easily reveals the typical structure of CMAC with its avascular “soft” centre, fibrotic capsule and the irregularly calcified rim (Fig. 1E, F) [14]. Tuberculoma are characterised by a hyper-dense core. Table 2 summaries the typical characteristics of the more common myxoma, intracardiac thrombus, abscess and tuberculoma with the three aforementioned imaging modalities. However, the pathognomonic depiction of CMAC described above requires pre- and post-contrast scans in CT, which involve significant radiation exposure for the patients. Nevertheless, MRI and CT images of CMAC resemble the macroscopic descriptions with a toothpaste-like consistency in the centre as an admixture of calcium, fatty acids, and cholesterol, which is not possible in Echo [6, 15]. The cases presented here clearly demonstrate the advantage of the systematic utilisation of all three imaging modalities in diagnosing CMAC in uncertain cases. Due to radiation exposure in CT and long measurement times in MRI, which could be burdensome for patients, we believe the use of CT and MRI should be reserved for cases where Echo does not provide a definitive CMAC diagnosis. However, multimodality imaging increasingly plays a major role in the planning of complex interventional valve treatments, as demonstrated in two of our cases. As our population continues to age, the atherosclerotic burden will increase, while traditional surgical strategies will become less feasible due to the increasing frailty of patients. Some clinical observations thus need to be mentioned: the majority of our patients with CMAC were females of advanced age, with pre-existing cardiovascular risk factors such as hyperlipidaemia with statin therapy and arterial hypertension with renal disease. Interestingly, these risk factors are known to be associated with MAC, but are not established in CMAC [16]. MAC, for instance, is an independent predictor of myocardial infarction and vascular death, while the clinical implications of CMAC are not fully understood [1, 5]. Further studies need to explore whether CMAC is the result or the expression of a chronic inflammatory activity with a calcium disorder separate from MAC [17]. Even the pathophysiological mechanism contributing to the formation of MAC needs further elucidation. This is all the more true for CMAC, an even rarer entity.

**Table 2:** Comparison of typical features of intracardiac masses in multimodality imaging

	CMAC	Myxoma	Thrombus	Abscess	Tuberculoma
Localisation/Morphology	Posterior MV annulus, smooth border	75% LA, interatrial septum, smooth	Ubiquitous, mainly LA, smooth	Ubiquitous: peri/valvular ≫ myocardial	Mainly pericardial or pervascular
Echogenicity	Overall: hyper, central areas: hypo	Hyper, inhomogeneous	Inhomogeneous	core: hypo border: hyper PA: blood flow	Inhomogeneous hyper
*MRI*					
T1-w intensity	Hypo	Iso	Subacute: hypo, chronic: hypo	Centre: hypo	Iso to hyper
T2-w intensity	Hypo	Hyper	subacute: hypo, chronic: hyper	Centre: hypo	Mainly iso
FP uptake	No	Heterogeneous	No	No	Heterogeneous
LGE uptake	Central: no Periphery: hyperenhancement (“rim”)	Heterogeneous	No Exception: chronic or organised thrombus may show peripheral ring enhancement—DHE	Hyper	Heterogeneous
CT density	Centre: hypo, no contrast enhancement, periphery: hyper → calcifications (bone window)	Iso, heterogeneous contrast enhancement	Hypo to iso, no contrast enhancement	Centre: hypo, periphery: contrast enhanced, PA: contrast filled	Centre: hyper → calcification (bone window)

CMAC, caseous mitral annular calcification; MRI, magnetic resonance imaging; T1-w, T1-weighted; T2-w, T2-weighted; FP, first pass; LGE, late gadolinium enhancement; CT, computed tomography; MV, mitral valve; LA, left atrium; DHE: delayed gadolinium hyperenhancement; PA: pseudoaneurysm

## Conclusion

CMAC is a rare condition, and most clinicians and even radiologists are unfamiliar with it. CMAC can be mistaken for an intra-cardiac tumour, vegetation, or abscess. Non-invasive multimodality imaging (i.e. Echo, MRI, and CT) in uncertain cases helps establish a definitive diagnosis of CMAC and avoid unnecessary interventions.

## Data Availability

All data generated or analyzed during this study are included in this published article.

## Abbreviations

CMAC: Caseous mitral annular calcification
MAC: Mitral annular calcification
Echo: Echocardiography
MRI: Cardiac magnetic resonance imaging
CT: Cardiac computed tomography
